# Centennial-scale reductions in nitrogen availability in temperate forests of the United States

**DOI:** 10.1038/s41598-017-08170-z

**Published:** 2017-08-10

**Authors:** K. K. McLauchlan, L. M. Gerhart, J. J. Battles, J. M. Craine, A. J. Elmore, P. E. Higuera, M. C. Mack, B. E. McNeil, D. M. Nelson, N. Pederson, S. S. Perakis

**Affiliations:** 1 Department of Geography, Kansas State University, Manhattan, Kansas 66506 USA; 2 Department of Environmental Science, Policy, and Management, University of California, Berkeley, California 94720 USA; 3 Jonah Ventures, LLC, Manhattan, Kansas 66502 USA; 4 University of Maryland Center for Environmental Science, Appalachian Laboratory, Frostburg, Maryland, 21532 USA; 5 Department of Ecosystem and Conservation Sciences, University of Montana, Missoula, Montana 59812 USA; 6 Center for Ecosystem Science and Society, Northern Arizona University, Flagstaff, Arizona 86011 USA; 7 Department of Geology and Geography, West Virginia University, Morgantown, West Virginia 26506 USA; 8 Harvard Forest, Harvard University, Petersham, Massachusetts 01366 USA; 9 U.S. Geological Survey, Forest and Rangeland Ecosystem Science Center, Corvallis, Oregon 97331 USA; 10 Department of Biology, University of Hawai’i, Mānoa, Honolulu HI 96822 USA

## Abstract

Forests cover 30% of the terrestrial Earth surface and are a major component of the global carbon (C) cycle. Humans have doubled the amount of global reactive nitrogen (N), increasing deposition of N onto forests worldwide. However, other global changes—especially climate change and elevated atmospheric carbon dioxide concentrations—are increasing demand for N, the element limiting primary productivity in temperate forests, which could be reducing N availability. To determine the long-term, integrated effects of global changes on forest N cycling, we measured stable N isotopes in wood, a proxy for N supply relative to demand, on large spatial and temporal scales across the continental U.S.A. Here, we show that forest N availability has generally declined across much of the U.S. since at least 1850 C.E. with cool, wet forests demonstrating the greatest declines. Across sites, recent trajectories of N availability were independent of recent atmospheric N deposition rates, implying a minor role for modern N deposition on the trajectory of N status of North American forests. Our results demonstrate that current trends of global changes are likely to be consistent with forest oligotrophication into the foreseeable future, further constraining forest C fixation and potentially storage.

## Introduction

N deposition rates to forests are generally elevated over pre-Industrial levels due to widespread increases in supplies of reactive N from human activities, potentially increasing forest N availability^1^. These increases in N deposition, along with changes observed in some ecosystems^2^, have led to the suggestion that a planetary boundary has been crossed, leading to wide-spread eutrophication that will destabilize the Earth system and human society^3, 4^. However, other global change factors, such as elevated CO_2_ can stimulate plant productivity and reduce N availability, despite increased deposition^5^. Ongoing environmental changes such as altered precipitation^6^, drought, and warming^7^ are also likely to directly affect N cycling although the total magnitude and direction of change are unknown. Therefore whether N availability has been increasing or decreasing—and over which spatial and temporal scales—is an open question. In support of the declining N hypothesis, observations of leaf N concentrations in European forests have shown multi-decadal declines^8^, forests of the eastern U.S. have demonstrated increased demand for N relative to supply^9^, and streamwater nitrate export has been declining for decades at sites in the U.S.^10^ and Europe^11^. Despite these indications, there is no coherent picture of the long-term changes in N availability of forested ecosystems at broad scales.

**Figure 1:**
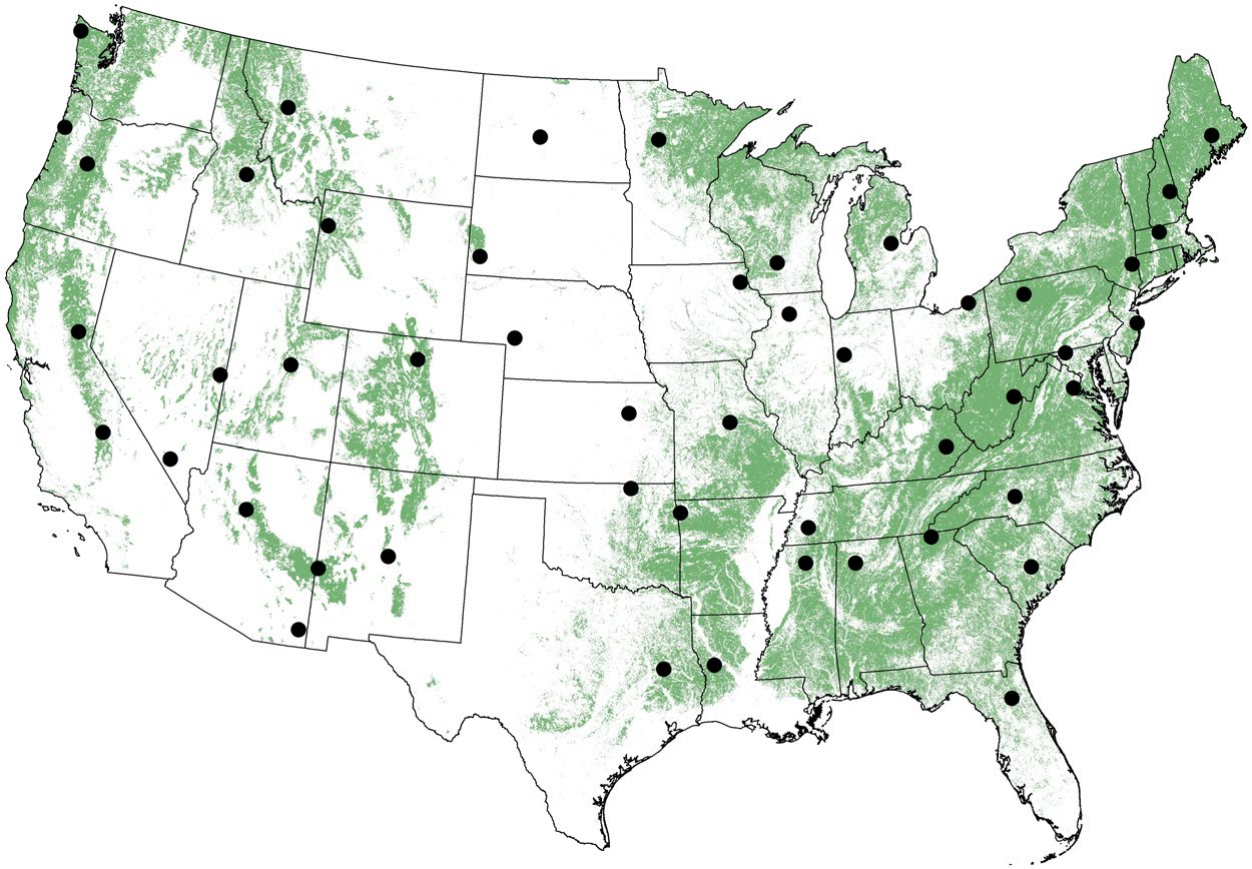
Locations of 49 sites sampled for wood δ^15^N in the continental U.S.A. Woody cover (green) is from the Commission for Environmental Cooperation. Full site information is available in Supplementary Table 1. Map was generated in ArcGIS 10.1 (https://www.arcgis.com/features/index.html).

Because it is unknown how the pace and trajectory of N availability will interact with multiple changes to the Earth system^12, 13^, current and future trajectories of forest N cycling are difficult to predict. Hence, there is considerable uncertainty about future forest productivity because it depends on availability of the limiting nutrient, N. As a critical step toward reconstructing historic trajectories in N availability for North American forests, we measured the isotopic composition of N stored in wood of living trees from a variety of forest types and climatic conditions. The stable N isotopic composition (δ^15^N) of plant tissue tends to increase with increasing N availability^14^ as sites with high N availability generally lose a high proportion of their N through fractionating loss pathways^15^. The δ^15^N of N acquired by plants is recorded in wood, which serves as a long-term, stable record of N availability experienced by trees^16^. Here, we assembled data from published literature and acquired new data on trajectories of δ^15^N in tree rings to allow for reconstruction of N availability for 49 forest sites across the continental United States (Fig. 1, Supplementary Table 1). Our samples comprise 309 total trees and 8394 wood δ^15^N values. The average length of the records is 123 y (s.d. ± 60 y), with an average first year of 1888 C.E. All sites had trees with wood dated from 1970 to 2005. A large portion of the climate space in North American forests is represented by our sampled sites, which range in mean total annual precipitation (MAP) by over 2400 mm, mean annual temperature (MAT) by 19 °C and N deposition by 14.5 kg ha^−1^ y^−1^ (Supplementary Fig. 1). Thus, our sample reflects much of the variation in forests across the U.S. and applies to a wide variety of forest types.

## Results

**Figure 2:**
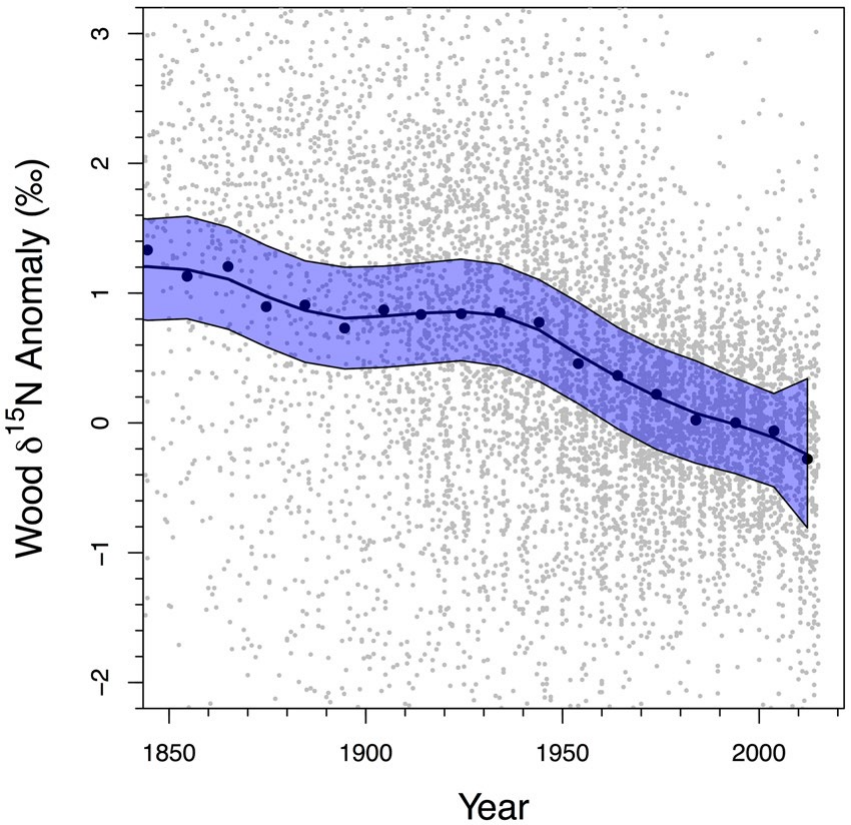
Changes in wood δ^15^N values since 1850 C.E. The change in wood δ^15^N anomaly averaged at the decadal scale. All wood δ^15^N data for each tree core was arithmetically adjusted to have a mean wood δ^15^N of 0‰ from 1970–2015 and then wood δ^15^N anomalies were averaged for each decade (black symbols). Gray symbols represent unsummarized individual adjusted wood δ^15^N values (N = 8934). Thin black lines and blue area represent 95% confidence interval for the loess-smoothed curve of wood δ^15^N values.

Trajectories of wood δ^15^N have been declining from 1850 C.E. to present, demonstrated when site-level patterns are summarized in a composite curve (Fig. 2). Data for each tree core were standardized so that the mean wood δ^15^N for the period after 1970 had a mean value of 0 (see Methods). An average wood δ^15^N value was then calculated for each site for each decade as well as the average year within the decadal range that the wood was sampled. When averaged across all sites, the composite curve reveals declining wood δ^15^N values over nearly the entire period of analysis. The cumulative change in wood δ^15^N from 1850 to 2010 was a decline of 1.41 ± 0.34‰, with 1.13 ± 0.34‰ of that occurring since the 1930s. These values are equivalent to an annual rate of change of −0.009‰ y^−1^and −0.014‰ y^−1^, respectively. This pattern did not change significantly when calculated using only the 45 sites that predate 1930 (92% of total sites); the cumulative change in wood δ^15^N for these sites over the entire record was equivalent to a decline in wood δ^15^N of 1.45 ± 0.34‰.

**Figure 3:**
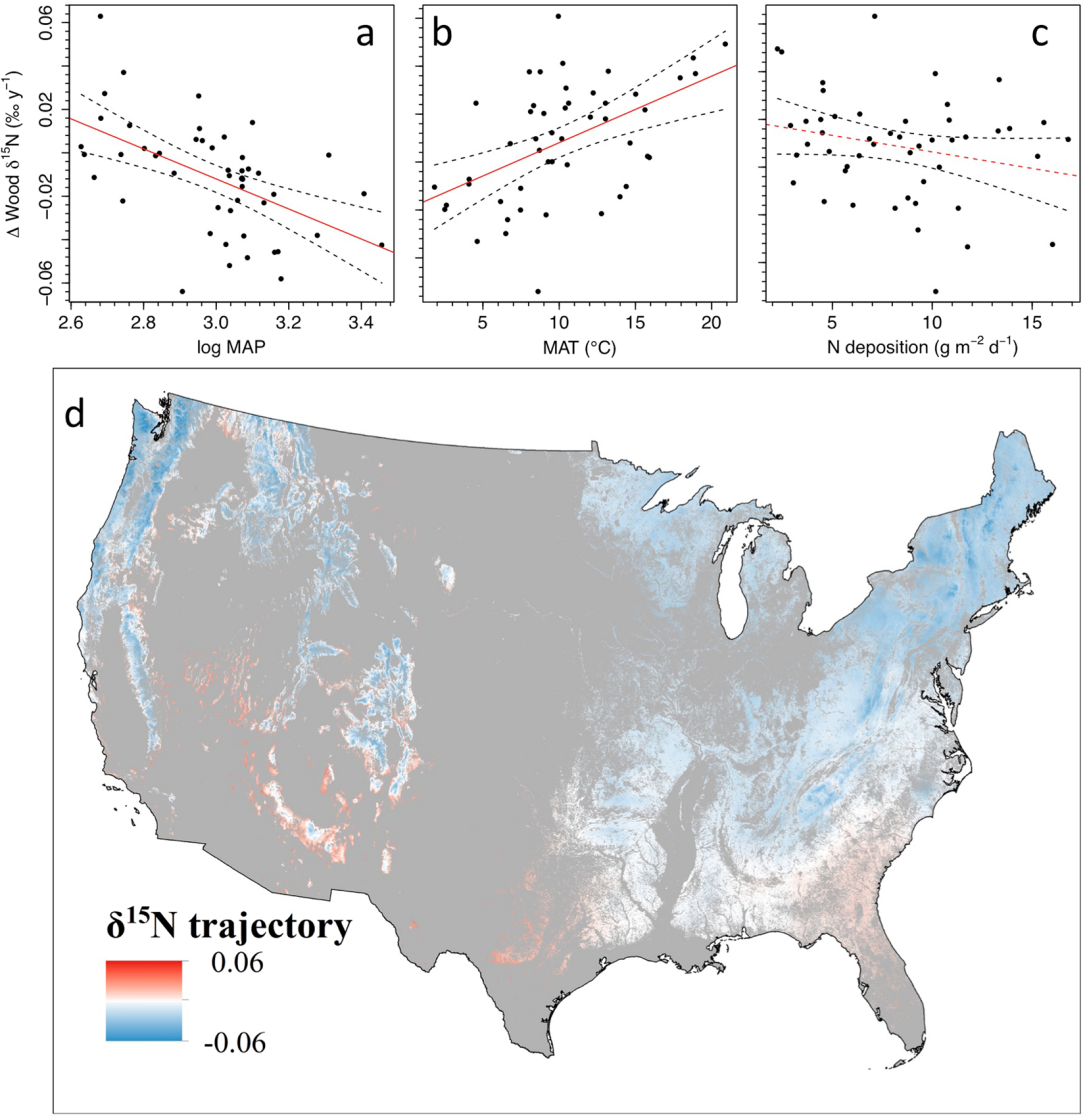
The effect of three site-specific variables on trajectories of wood δ^15^N since 1970 C.E. (**a**) log of mean annual precipitation (estimate −0.069 ± 0.018, *P* < 0.001) (**b**) mean annual temperature (estimate 0.0028 ± 0.0008, *P* < 0.001) (**c**) atmospheric N deposition (estimate −0.0014 ± 0.0009, *P* = 0.12). (**d**) map of modeled wood δ^15^N trajectories since 1970 C.E. using these regression relationships for forested areas in the U.S. Units are ‰ y^−1^. Data source for forested area: Commission for Environmental Cooperation. For panel d, the projection of δ^15^N trends was generated by using free software R (R Core Team (2013). R: A language and environment for statistical computing. R Foundation for Statistical Computing, Vienna, Austria. URL: https://www.R-project.org/) and the map was generated in ArcGIS 10.1.

The geographic patterns of declining wood δ^15^N can be analyzed in more detail over the past few decades because the time period covered by all sites begins in 1970 (Supplementary Fig. 2). Individual sites vary in their trajectories from an increase of 0.051‰ y^−1^ to a decrease of 0.069‰ y^−1^ from 1970 to 2014. Linear regressions of wood δ^15^N trajectories over time since 1970 indicated that 33 of the 49 sites showed declines in wood δ^15^N, with the remainder showing increases (Supplementary Fig. 3). Significance was not tested for each site-level regression. Climate variables were correlated with the recent trajectories of wood δ^15^N (Fig. 3). The decline toward present in wood δ^15^N was most pronounced in cool, wet forests. For example, the model estimated that forests with a MAP = 1000 mm had declined in wood δ^15^N by 0.48‰ over a 40-year period, while those with MAP = 1500 mm were estimated to have declined by 0.95‰ (Fig. 3A). Although the model suggests that dry sites have increasing wood δ^15^N toward present, this trajectory is only significant for the very driest sites (MAP < 435 mm). Cooler forests declined in δ^15^N at a faster rate than warmer forests (*P* = 0.001, Fig. 3B). The magnitude of declines across the MAT gradient was the equivalent of a difference of 2.0‰ in wood δ^15^N over 40 years between the forests with coolest and warmest MAT, when compared at mean MAP and N deposition rates. Despite strong patterns of trajectories in wood δ^15^N, spatial variation in levels of N deposition had no significant effect on the trajectory of wood δ^15^N among sites (Fig. 3C, *P* = 0.13). The lack of a significant effect of N deposition occurred whether rates of change in wood δ^15^N were calculated beginning in 1960 or 1970 (*P* > 0.12 for both).

We used a random effects model to test whether growth rates and tree functional group were influencing wood δ^15^N trajectories. Both mean basal area increment (BAI) and the slope of BAI over time (both since 1970) were regressed against the residuals of the model of modern wood δ^15^N. Mean BAI since 1970 varied among sites by over 3700 mm^2^ y^−1^ (central 95% of the values = 65 to 3811 mm^2^ y^−1^). Individual trees also varied greatly in their trajectory of BAI over time, with the central 95% of the values ranging from −82 mm^2^ y^−2^ to 52 mm^2^ y^−2^. After accounting for climate and N deposition, there was no influence of wood production or trajectories in wood production on the change in wood δ^15^N over time. Neither sites with greater BAI nor sites that were increasing in BAI the most were declining in δ^15^N more than others (*P* > 0.37 for both). Likewise, the age of trees at each site did not influence the rate or magnitude of declines in wood δ^15^N (*P* = 0.69). A parallel mixed-effects regression model examined relationships between wood δ^15^N and predictors for individual trees and indicated no significant differences in the declines in wood δ^15^N between angiosperms and gymnosperms (*P* = 0.40, Supplementary Table 2). Within a site, however, there are examples of different wood δ^15^N trajectories among tree species^17, 18^.

We used the results of the linear regressions to extend the spatial patterns of trajectories of wood δ^15^N to all forested sites in the U.S. for the past 40 years (Fig. 3D). We used fine-scale datasets of MAP and MAT to extrapolate the past trajectories of wood δ^15^N. Applying our regression coefficients to estimate the trajectory of wood δ^15^N over time suggests 84.5% of the forested locations in the U.S., equivalent to 172.5 Mha, have likely been experiencing declining N availability. Only the warmest, driest forests such as those in the southwestern U.S. are most likely to be experiencing increased N availability over time. It remains to be seen if the relationships between N availability and climate we found across space are similar to those associated with changes in climate over time. Although some forested regions of the U.S. have been getting wetter^5^, and the patterns found here are similar to recent changes in hydroclimate, future climate scenarios project warmer and drier climate conditions in most forested regions of the U.S.^19^. Both moisture and temperature certainly affect N cycling processes^6^. Thus, it will be important to assess the quantitative nature of projected associated changes in N cycling over time^20^.

## Discussion

Although many aquatic and even some terrestrial ecosystems in the U.S. are showing evidence of eutrophication^2, 21^, forests generally appear to be experiencing oligotrophication^22^, which is consistent with previous analyses of temporal trends in declining δ^15^N values in other ecosystem compartments such as leaves^23^ and sediments^24^ as well as foliar N concentrations^8^ and observed declines in streamwater N export^10, 11^. Similarly, trends in declining wood δ^15^N have been observed at the single site^17, 18, 25^ to sub-regional^9^ level in eastern North America and attributed to various causes. The proximal reasons why wood δ^15^N are declining are uncertain at this point, but potentially involve a reduction in relative denitrification rates or nitrification rates, or an increase in reliance on mycorrhizal fungi for N acquisition. Our study provides an important link between the site-level and global-level^26^ patterns in N cycling.

More distally, based on three lines of evidence, it is unlikely that the general declines over the past century in wood δ^15^N are being caused either by plant uptake of atmospherically-deposited, ^15^N-depleted N or significant alterations to the N cycle caused by N deposition. First, the recorded changes in the N cycle predate invention of the Haber-Bosch process in 1910 and certainly the onset of widespread inorganic N fertilizer use in the U.S. in the mid-1900s^27^. Second, there was no discernable role of N deposition in determining mean values or temporal trends in site-level wood δ^15^N trajectories. Third, although there appears to be no characteristic isotopic signature of atmospherically-deposited N, the quantity of N deposited and its δ^15^N values are positively correlated^28^, so high quantities of N deposition would be unlikely to reduce wood δ^15^N as observed here.

The timing and coherence of observed declines in wood δ^15^N implicates a powerful driver acting over a large spatial extent as early as 1850. One potential hypothesis is that increased atmospheric CO_2_ concentrations are influencing the wood patterns observed here. Increased atmospheric CO_2_ concentrations have been demonstrated to reduce N availability in terrestrial ecosystems^7, 13, 29^. Given that N is often the most important nutrient limiting temperate forest productivity^30^, reduced C fixation and primary productivity relative to predicted levels are more likely in the future^31^. Although there remains a need for better constraints on other important aspects of the N cycle, such as N_2_ fixation^32^ and denitrification^33^, there is little to indicate that forest N availability will increase in the near future. N deposition rates are generally declining in North America^34^, atmospheric CO_2_ concentrations continue to increase, and projected changes in temperature and precipitation^19^ are likely insufficient to reverse the trend in declining N availability. As climate continues to change, N availability will determine the balance between ecosystem productivity and potentially negative environmental consequences of excess N. Our results are consistent with a long-term decline in forest N loss and are relevant for downstream riverine and coastal systems, which are experiencing increased N loading from agricultural and urban sources^35^. With over 30% of the continental U.S. currently in forested land cover, understanding the long-term changes in N cycling, and ultimately the drivers of these changes in N cycling, remains an important challenge.

## Methods

### Sample collection

At each of 39 sites sampled, one increment core was taken from each of 10 individual trees using a 5.15-mm diameter increment borer (Haglöf). Sites were selected to be geographically distributed within the contiguous United States, represent a natural or semi-natural area, contain over 50% forested canopy cover, and span a wide range of climates. Tree selection at each site targeted individuals in canopy positions with straight stems, from species representative of the overall stand composition. We therefore sampled multiple tree species at sites with mixed species assemblages. Cores that were damaged, appeared to be stained from fungal colonization, or contained fewer than 40 consecutive annual rings were excluded from further consideration.

### Ring-width analysis

Following sampling, cores were dried for at least 72 h at 70 °C, then sanded using 400 grit sandpaper. After sanding, each core was scanned at 1600 dpi using an Epson Expression 1000 XL scanner (Epson Corporation, Long Beach, CA). From these high-resolution images, the widths of all individual rings were quantified using Cybis CooRecorder v7.8 and CDendro v7.8 (Saltsjöbaden, Sweden). Although we performed tests of ring-width coherence for all trees within a site, some dating imprecision likely remains with these chronologies, and therefore we selected analytical and statistical approaches that allowed for integration of wood δ^15^N data at super-annual timescales.

### Nitrogen isotope analysis

For each site, cores from at least three trees were analyzed for wood δ^15^N, generally selecting the longest series for analysis. First, increment cores were sectioned by hand with a razor blade along ring boundaries until the sample approximated a target weight of 30 mg. Ultimately, individual rings were divided or aggregated into 25–30 mg segments, providing enough N for isotopic analysis while limiting the carbon content and the risk of incomplete combustion. On average, each sample represents 1.6 years of wood. Not all samples of wood were analyzed for δ^15^N; we selected enough samples from each core to provide at least two measurements of wood δ^15^N per decade. No chemical pretreatment of wood samples was performed as none of the reported pretreatment protocols consistently identify and remove labile N components in wood (see ref. 16 for a discussion of pretreatment protocols). Due to potential inter-ring mobility of N-containing compounds^36^, we compiled and analyzed our data at super-annual timescales.

After partitioning, wood tissue was wrapped in pressed tin capsules and analyzed at one of six labs: the University of California Davis Stable Isotope Facility using a PDZ Europa ANCA-GSL elemental analyzer interfaced to a PDZ Europa 20–20 isotope ratio mass spectrometer (Sercon Ltd., Cheshire, UK), the University of New Mexico Stable Isotope Laboratory using a a Costech 4010 elemental analyzer (Valencia, CA) connected to a ThermoFinnigan Delta V isotope ratio mass spectrometer (Bremen, Germany), the Central Appalachians Stable Isotope Facility at the University of Maryland Center for Environmental Science, the Stable Isotope Mass Spectrometry Laboratory at Kansas State University, the University of Florida, and the W. M. Keck Paleoenvironmental and Environmental Stable Isotope Laboratory at the University of Kansas. All elemental analyzers employed traps of MgClO_4_ and Carbosorb to absorb H_2_O and CO_2_, respectively. All samples for any given core were analyzed at only one of the locations. The standardized ratio of ^15^N:^14^N is reported using delta notation (δ^15^N) in per mil format (‰) relative to AIR. Each lab used a two-point normalization curve with internal standards calibrated against international standards.

In addition to the 39 sites collected *de novo* here, additional wood δ^15^N data were complied from the published literature (three sites) and unpublished data (seven sites). The criteria for inclusion of these data included the following requirements: (1) δ^15^N data must be available for a minimum of three trees per site, (2) the δ^15^N chronologies must extend at least 40 years, and (3) data must have been collected since 2005.

### Site environmental covariates

For each site, mean annual temperature (MAT) and mean total annual precipitation (MAP) were obtained from the PRISM Climate Group (http://prism.oregonstate.edu) incorporating interpolated data averaged from 1981–2010. Mean annual atmospheric N deposition was obtained from the National Atmospheric Deposition Program National Trends Network (NTN) monitoring stations. Total N deposition (wet + dry) averaged from 2000–2013 for each site was extracted from deposition maps provided by the NADP at http://nadp.sws.uiuc.edu/committees/tdep/tdepmaps/. Although climate variables and N deposition levels are dynamic during the duration of the tree-ring record, including the period of analysis 1970-present, we decided to use a summary of the most accurate data for predictor variables for this time period. The assumption with this approach is that across space, relative values of climate variables and N deposition have remained similar. For the time periods for which we have N deposition data and climate data, that assumption is supported.

### Data analysis

To assess recent patterns of N cycling in forests, the slope of wood δ^15^N from 1970 to the end of the record was calculated for each core at a site using a simple linear regression. Site-level mean slopes were then calculated and relationships with log-transformed MAP, MAT, and N deposition were assessed with a multiple regression. No outliers were detected in the regression model, based on a Bonferroni Outlier Test (*P* > 0.05). Residual wood δ^15^N from this model was regressed against mean basal area increment (BAI) since 1970 and the linear slope of BAI over time since 1970. BAI was calculated from the inside of the core outwards based on ring-width measurements. Basal Area Increment (*BAI*_*t*_) was calculated for each year as follows:

$$BA{I}_{t}=\pi {{r}_{t}}^{2}-\pi {{r}_{t-1}}^{2}$$

where *r*_*t*_ = radius of outside of ring, and r_0_ = 0.

To determine whether there were differences between angiosperms and gymnosperms in their trajectory of wood δ^15^N over time, a random effects model that paralleled the multiple regression model was assessed using the slopes from individual trees of wood δ^15^N since 1970, but included the species’ taxonomic group (gymnosperm vs. angiosperm) and site identity as a random effect in addition to MAT, MAP, and N deposition. There were not enough samples of any tree species to allow for species-level analysis. Twelve of the 49 sites had more than one tree taxonomic group (gymnosperms and angiosperms). Our study design did not target tree taxonomic group, rather we focused on sampling representative trees at each site.

To assess the long-term patterns of wood δ^15^N, the average wood δ^15^N value from 1970–2015 was calculated for each increment core. A constant that was opposite in sign to this average was then added to each wood δ^15^N value for the increment core. This standardizes the average wood δ^15^N value from 1970–2015 = 0‰ and all individual values to represent an anomaly from this standardized mean. Data for all cores were then averaged at the decadal scale for each site and a second decadal-scale average generated across all sites. A loess curve was then fit to these data and curves representing the 95% confidence interval for the predicted fit was calculated. This is a common approach used to synthesize spatio-temporal data^37–39^.

All data analyses were conducted in R v. 3.2.5. Data for this manuscript are available at the Dryad Digital Repository.

## Electronic supplementary material

Supplementary Information
