# Supplementary material for: Centennial-scale reductions in nitrogen availability in temperate forests of the United States
Source: Sci Rep. 2017 Aug 10;7:7856. doi: 10.1038/s41598-017-08170-z (PMC5552780; doi:10.1038/s41598-017-08170-z)
Supplement: Supplementary file 1 — Supplementary Information [file 41598_2017_8170_MOESM1_ESM.pdf]

Supplementary Information

Centennial-scale reductions in nitrogen availability in temperate forests of the United States

K. K. McLauchlan, L. M. Gerhart, J. J. Battles, J. M. Craine, A. Elmore, P. E. Higuera, M. C. Mack, B. E. McNeil, D. M. Nelson, N. Pederson, S. S. Perakis

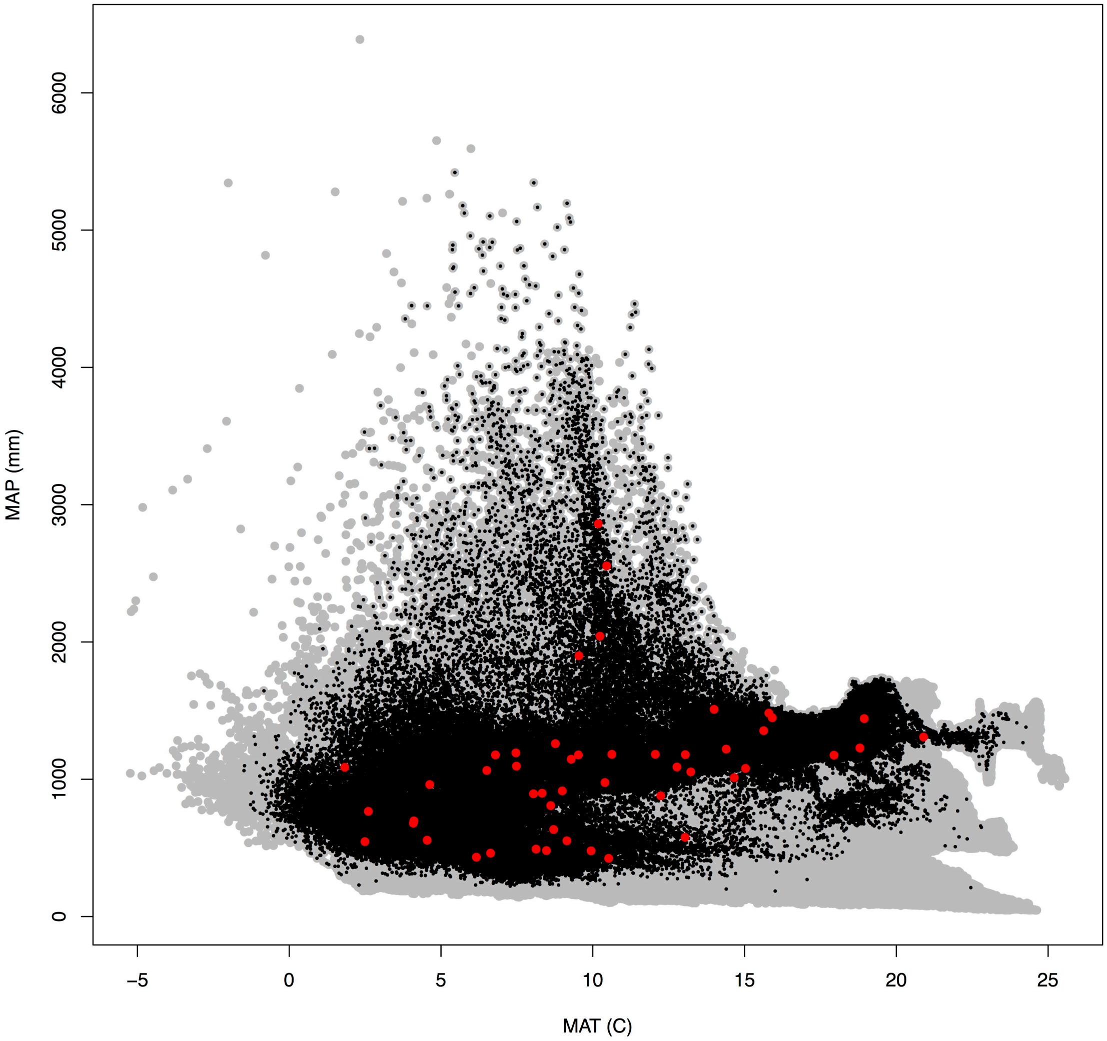

Supplementary Fig. 1| Climate space of forests in North America and forests sampled. Mean annual precipitation and mean annual temperature values for the conterminous U.S. (grey), all forested areas in the U.S. defined by the Commission for Environmental Cooperation (black), and our 49 sampled sites (red).

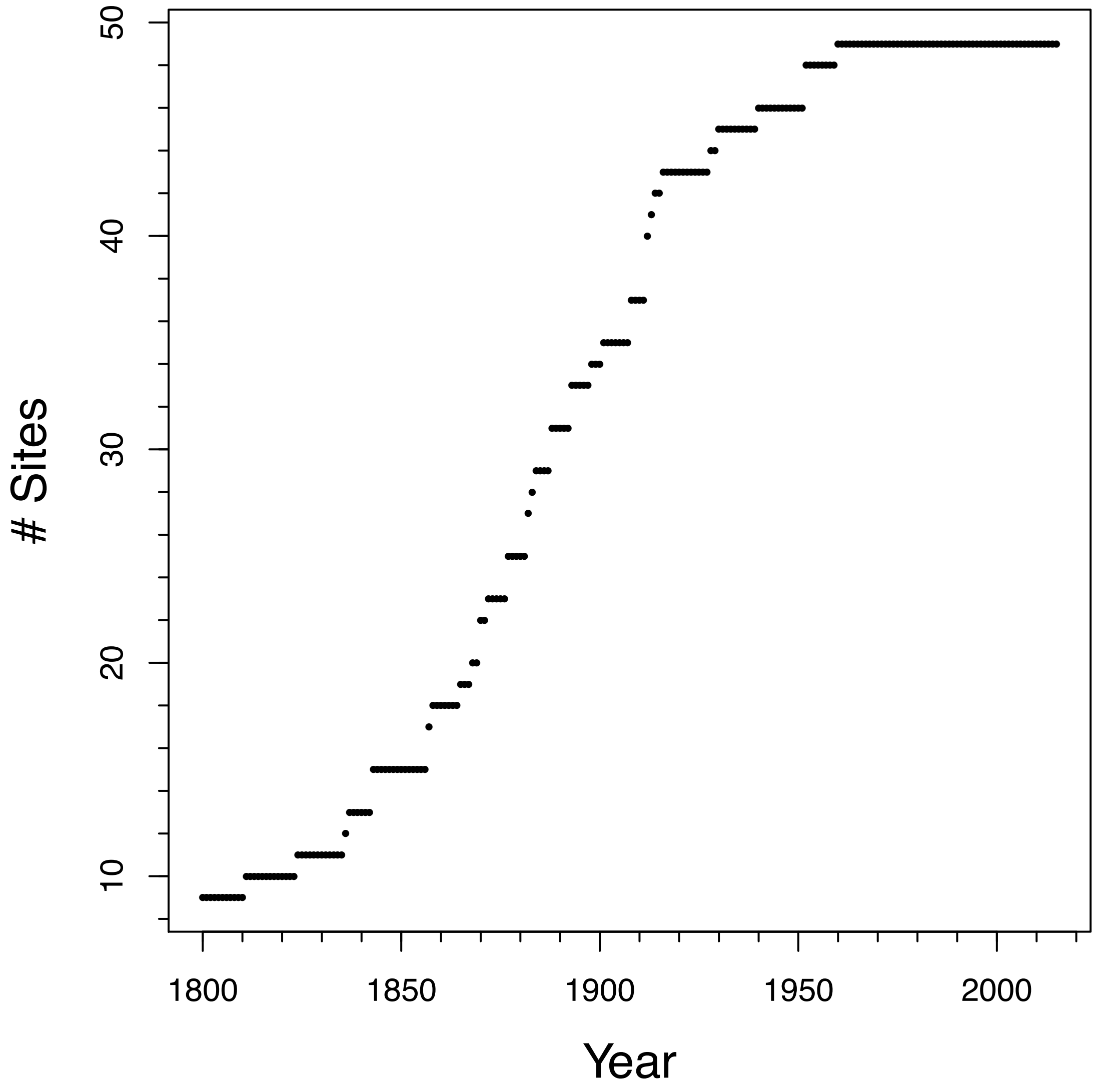

Supplementary Fig. 2| Number of sites with wood $\delta^{15}N$ samples prior to a given year.

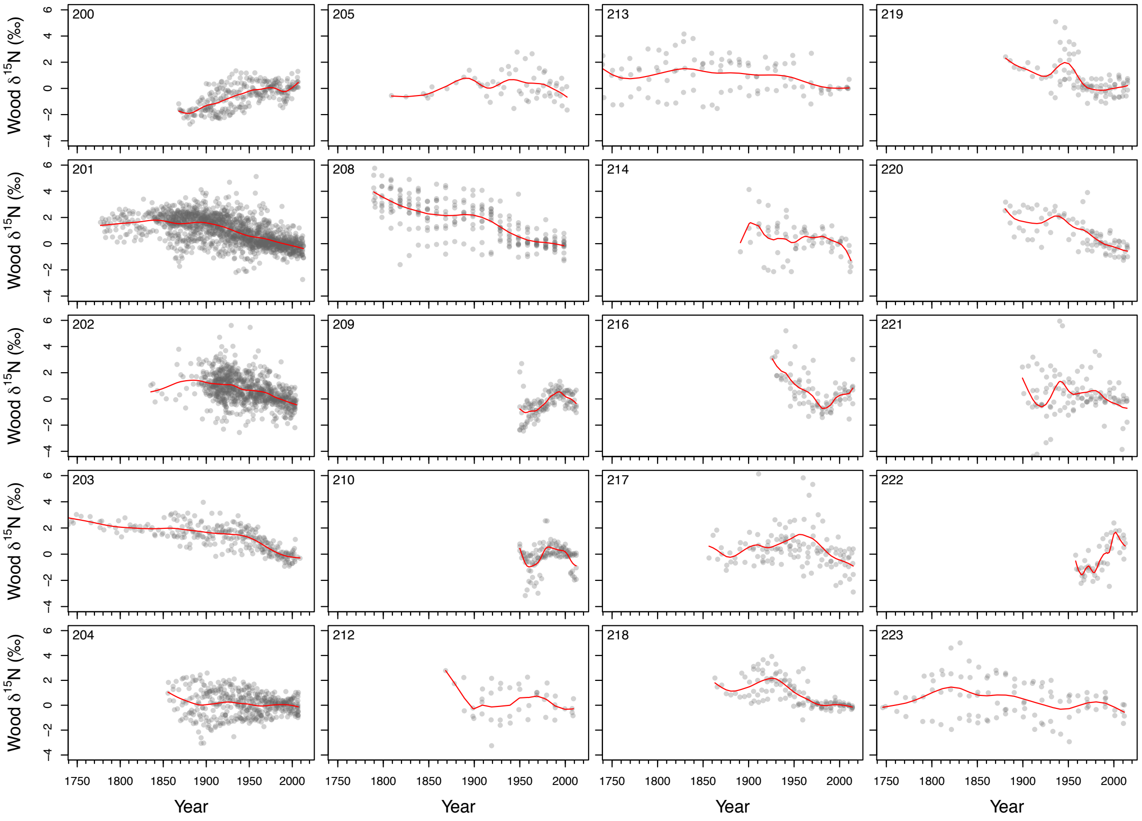
200
205
213
219
201
208
214
220
202
209
216
221
203
210
217
222
204
212
218
223
Wood δ15N (‰)
Year
1750 1800 1850 1900 1950 2000
-4 -2 0 2 4 6

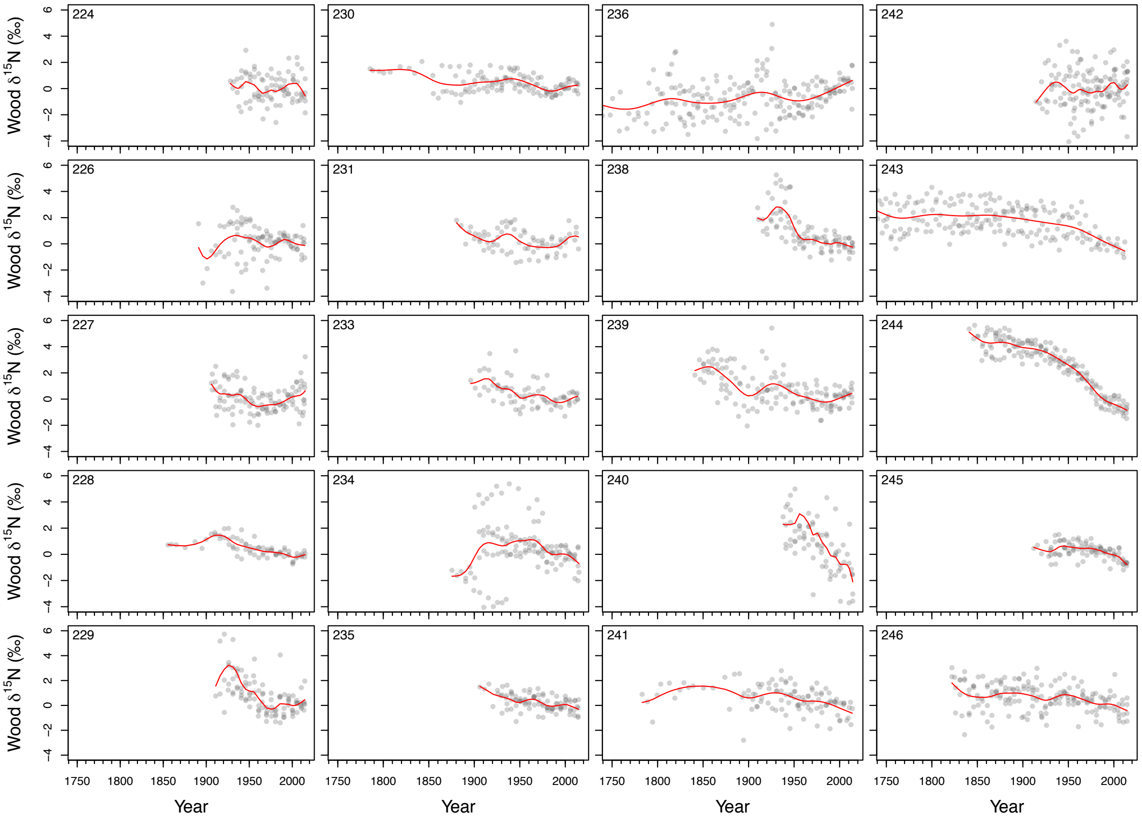
224
230
236
242
226
231
238
243
227
233
239
244
228
234
240
245
229
235
241
246
Wood δ15N (‰)
Year
1750 1800 1850 1900 1950 2000
-4 -2 0 2 4 6

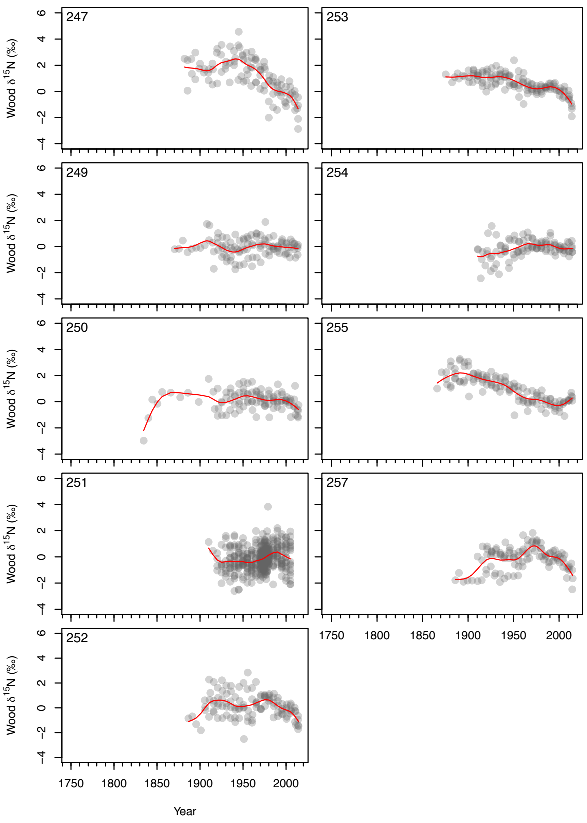

Supplementary Fig. 3| Wood $\delta^{15}N$ values from each of 49 individual sites. Data are standardized for each individual core so that measured values from 1970 to the most current samples average 0‰. Red line is a smoothing spline fit. Site ID code is a three-digit number in the upper left of each panel. Site ID metadata are in Supplementary Table 1.

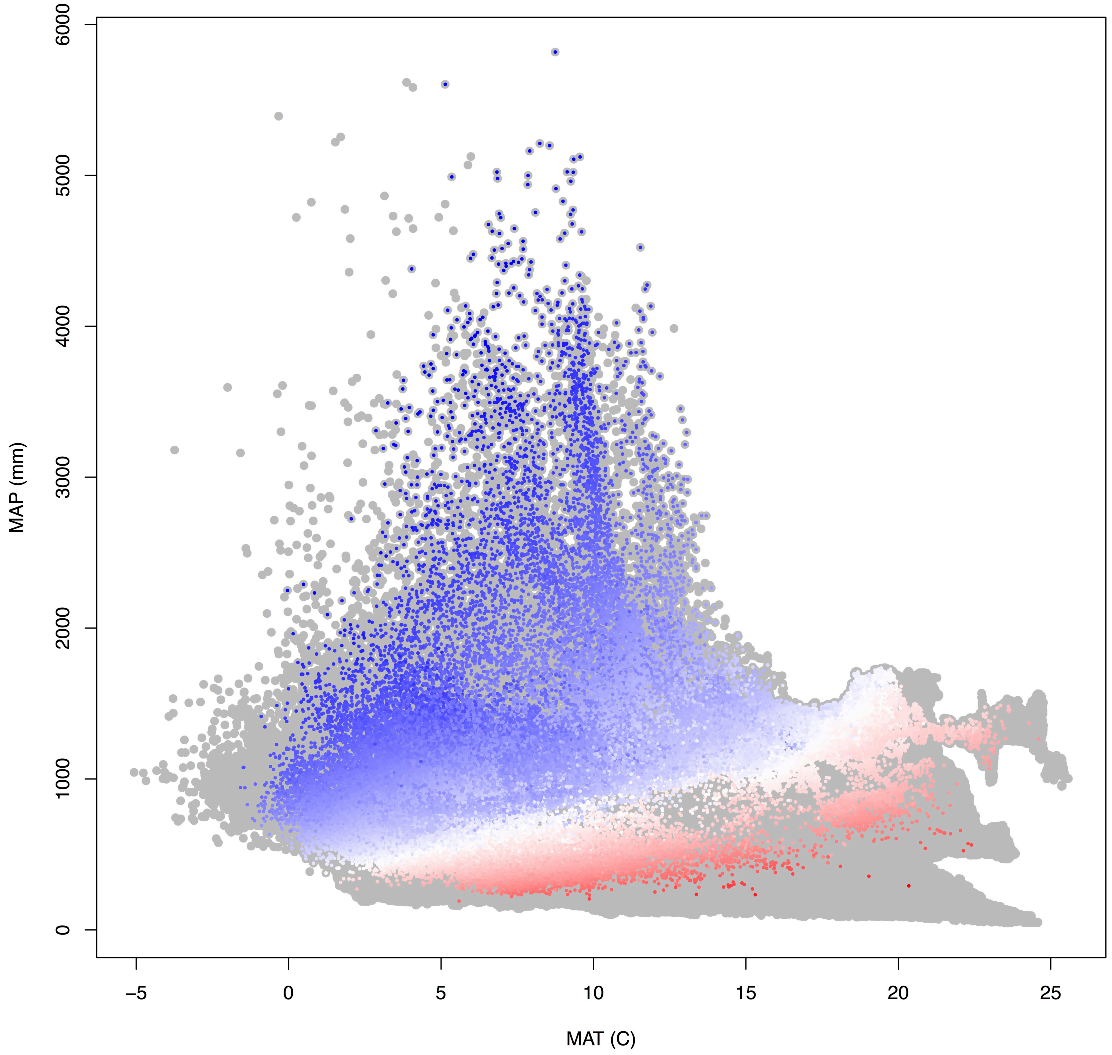

Supplementary Fig. 4| Projected trajectories mapped onto climate space. Mean annual precipitation and mean annual temperature values for the conterminous U.S. (grey) with our modeled trajectories of wood $\delta^{15}N$ based on statistical regressions with these climate variables. Blue indicates decreasing wood $\delta^{15}N$ values toward present; red indicates increasing wood $\delta^{15}N$ values toward present. Full color legend is available in Fig. 3D.

Supplementary Table 1| Metadata for each of the 49 sites used in this study. MAP = Mean Annual Precipitation (mm), MAT = Mean Annual Temperature (°C), N dep = N deposition (kg ha^{-1}). *indicates previously-published data by coauthors of this manuscript †indicates unpublished data collected by coauthors prior to the collection effort for this manuscript

Site Name	State	Site ID	Lat	Long	Genera	Elevation (m)	MAP	MAT	N dep	Oldest Year	Chron. length
Bankhead NF	AL	214	34.31	-87.39	*Pinus, Quercus, Tsuga*	222	1482	15.8	11.31	1891	118
Wedington Gap	AR	244	36.1	-94.39	*Quercus*	392	1220	14.4	16.03	1841	116
Coal Pit Canyon	AZ	231	31.86	-109.37	*Pinus*	1835	578	13.04	5.13	1880.5	123
Dogtown Lake	AZ	233	35.2	-112.17	*Juniperus, Pinus*	2218	634	8.71	4.5	1896	123
Sequoia, King's Canyon NP†	CA	205	36.58	-118.58	*Abies*	2833	963	4.63	8.14	1809	115
Tahoe NF	CA	252	39.32	-120.64	*Abies, Calocedrus, Pinus*	1588	1900	9.55	6.35	1886.5	112
Chickaree Lake†	CO	208	40.33	-105.86	*Pinus*	2806	547	2.5	4.84	1789.5	182
Ordway-Swisher	FL	216	29.69	-81.97	*Pinus*	43	1309	20.9	6.37	1926	83
Chattahoochee NF	GA	217	34.85	-84.24	*Quercus*	550	1510	14	9.3	1856.5	125
Big Springs Fish Hatchery	IA	218	42.91	-91.47	*Carya, Juglans, Quercus*	293	900	8.33	15.59	1863	122
Mountain Ecosystem Sensor Array (MESA)	ID	212	45.01	-114.84	*Pseudotsuga*	1795	1088	1.83	3.69	1868.5	132
Nachusa Grasslands	IL	255	41.88	-89.34	*Quercus*	240	916	9	13.88	1866	142
Southern Great Lakes Forest*	IN	204	40.54	-87.05	*Acer, Carya, Fagus, Quercus*	231	976	10.4	13.3	1855.5	107
Konza Prairie LTER†	KS	200	39.07	-96.54	*Quercus*	412	881	12.24	10.86	1868	96
Bucklick-Robinson Forest	KY	235	37.46	-83.15	*Acer, Liriodendron, Quercus*	362	1181	13.05	9.34	1906	90
Kisatchie NF	LA	219	31.49	-93.19	*Fagus, Pinus, Quercus*	58	1443	18.95	8.38	1881	91
Harvard Forest	MA	257	42.53	-72.19	*Quercus*	272	1193	7.47	8.79	1886	103
Catoctin Mountain†	MD	210	39.64	-77.45	*Liriodendron, Quercus*	383	1181	10.63	16.81	1950	62
Penobscot Experimental Forest	ME	247	44.88	-68.65	*Tsuga*	34	1064	6.51	4.59	1882	105
Veterans Memorial Park	MI	240	43.68	-84.4	*Pinus*	200	808	8.62	10.18	1938	77
Itasca*	MN	201	47.22	-95.15	*Pinus*	473	681	4.09	7.08	1781	119
Baskett Research Center	MO	221	38.75	-92.2	*Quercus*	226	1089	12.78	11.77	1899.5	112
U-Miss Field Station	MS	220	34.43	-89.39	*Pinus, Quercus*	135	1450	15.92	9.17	1880.5	85
Lolo NF	MT	236	47.21	-113.49	*Pseudotsuga*	1335	556	4.55	2.47	1572.5	288
Piedmont	NC	224	35.7	-80.62	*Pinus, Quercus*	213	1079	15.03	11	1928	79
Cross Ranch State Park	ND	254	47.22	-100.99	*Quercus*	522	433	6.17	5.66	1910.5	108
Cedar Point BFS	NE	222	41.21	-101.66	*Juniperus, Tilia*	994	480	9.95	7.13	1958	52
Mirror Lake*	NH	202	43.62	-71.25	*Acer, Betula, Fagus, Fraxinus, Tsuga*	178	1096	7.49	5.75	1835.5	106
Cedar Bridge	NJ	238	40.06	-74.13	*Pinus*	9	1183	12.06	9.99	1910	99
Cerro Montosa, Sevilleta LTER	NM	230	34.4	-106.21	*Juniperus, Pinus*	2081	425	10.53	3.21	1785.5	151
Luna	NM	239	33.78	-108.93	*Pinus*	1835	481	8.48	4.42	1841	166
Great Basin	NV	249	39.01	-114.25	*Abies*	2530	461	6.63	3.06	1870.5	108
Red Rock Canyon	NV	223	36.26	-115.62	*Pinus*	2520	554	9.15	6.04	1746	232

Cary Institute	NY	245	41.79	-73.73	*Quercus*	165	1146	9.29	10.36	1912	90
Willoughby	OH	234	41.61	-81.29	*Acer, Fagus*	285	1179	9.53	15.28	1875.5	121
Tallgrass Prairie Preserve†	OK	203	36.84	-96.45	*Quercus*	333	1012	14.66	9.57	1729	168
HJ Andrews Forest LTER	OR	213	44.21	-122.26	*Pseudotsuga*	751	2044	10.24	2.26	1633	303
Salmon River	OR	246	45.06	-123.75	*Pseudotsuga*	220	2555	10.46	4.52	1822	182
Kane Experimental Forest	PA	253	41.59	-78.77	*Acer, Fagus, Tsuga*	593	1179	6.8	11.69	1875.5	124
Santee NWR	SC	226	33.53	-80.43	*Pinus*	24	1176	17.95	7.91	1891	97
Wind Cave NP	SD	227	43.56	-103.48	*Pinus*	1298	493	8.13	4.54	1906	108
Hatchie NWR	TN	228	35.49	-89.17	*Quercus*	97	1355	15.63	8.89	1855.5	120
Davy Crockett NF	TX	229	31.4	-95.15	*Pinus*	95	1228	18.8	8.66	1911	99
Manti La Sal NF	UT	250	39.67	-111.32	*Abies, Populus*	2663	698	4.11	6.87	1834.5	107
Prince William Forest†	VA	209	38.57	-77.4	*Liriodendron, Quercus*	84	1053	13.23	10.76	1950	62
Hoh Forest	WA	241	47.95	-124.38	*Tsuga*	103	2862	10.18	2.91	1783	137
Devil's Lake	WI	242	43.43	-89.74	*Pinus*	361	895	8.05	13.35	1914.5	81
Monongahela NF†	WV	251	38.63	-79.98	*Liriodendron, Picea*	781.5	1260	8.77	10.15	1910	105
Grand Tetons NP	WY	243	43.94	-110.64	*Pinus*	2254	766	2.61	3.76	1611.5	291

Supplementary Table 2| Random effects model results

	Estimate	Std Error	P value
(Intercept)	0.174	0.054	0.001
log.MAP	-0.067	0.019	<0.001
MAT	0.003	0.001	0.002
Ndep	-0.002	0.001	0.09
FxnlGrp*	0.007	0.006	0.23

*estimate is for deciduous trees.
